# Learning improvement after PI3K activation correlates with *de novo* formation of functional small spines

**DOI:** 10.3389/fnmol.2013.00054

**Published:** 2014-01-02

**Authors:** Lilian Enriquez-Barreto, Germán Cuesto, Nuria Dominguez-Iturza, Elena Gavilán, Diego Ruano, Carmen Sandi, Antonio Fernández-Ruiz, Gonzalo Martín-Vázquez, Oscar Herreras, Miguel Morales

**Affiliations:** ^1^Structural Synaptic Plasticity Lab, Center for Biomedical Research of La RiojaLogroño, La Rioja, Spain; ^2^Department of Biochemistry and Molecular Biology, Neuroscience Institute, Universitat Autònoma de BarcelonaBarcelona, Spain; ^3^Instituto de Biomedicina de Sevilla, Universidad de SevillaSevilla, Spain; ^4^Brain Mind Institute, École Polytechnique Fédérale de LausanneLausanne, Switzerland; ^5^Experimental and Computational Electrophysiology Lab, Instituto Cajal, Consejo Superior de Investigaciones CientíficasMadrid, Spain

**Keywords:** dendritic spines, structural plasticity, PI3K, hippocampus

## Abstract

PI3K activation promotes the formation of synaptic contacts and dendritic spines, morphological features of glutamatergic synapses that are commonly known to be related to learning processes. In this report, we show that *in vivo* administration of a peptide that activates the PI3K signaling pathway increases spine density in the rat hippocampus and enhances the animals’ cognitive abilities, while *in vivo* electrophysiological recordings show that PI3K activation results in synaptic enhancement of Schaffer and stratum lacunosum moleculare inputs. Morphological characterization of the spines reveals that subjecting the animals to contextual fear-conditioning training *per se* promotes the formation of large spines, while PI3K activation reverts this effect and favors a general change toward small head areas. Studies using hippocampal neuronal cultures show that the PI3K spinogenic process is NMDA-dependent and activity-independent. In culture, PI3K activation was followed by mRNA upregulation of glutamate receptor subunits and of the immediate-early gene Arc. Time-lapse studies confirmed the ability of PI3K to induce the formation of small spines. Finally, we demonstrate that the spinogenic effect of PI3K can be induced in the presence of neurodegeneration, such as in the Tg2576 Alzheimer’s mouse model. These findings highlight that the PI3K pathway is an important regulator of neuronal connectivity and stress the relationship between spine size and learning processes.

## INTRODUCTION

Spines are micrometric protrusions of dendritic membranes. Their correlation with learning and memory has been suggested ever since their early description by Ram’o (1899). Nowadays, it is commonly accepted that spine size and density are features linked to the memory formation process (Bourne and Harris, 2007).

Spines are dynamic structures that come in a wide assortment of shapes and sizes (Harris and Stevens, 1989). An important feature of spine functionality is that they serve as compartments for glutamatergic receptors, the composition of which correlates with the spines’ head volume and physiological role (Alvarez and Sabatini, 2007). Accordingly, small spines are highly motile, capable of rapid expansion, with NMDA being the predominant receptor, whereas large spines are highly stable and have a predominance of AMPA receptors (Matsuzaki et al., 2001; Alvarez et al., 2007).

There is a large body of evidence indicating that sensorial experience alters spine density and shape. Rodents raised in an enriched environment display an increase of mossy fiber synapses through Wnt signaling (Gogolla et al., 2009), whereas mice trained to perform a forelimb motor task develop spines in the contralateral motor cortex area (Xu et al., 2009). Aging is another factor that modulates spine distribution; older monkeys exhibit a 35% reduction in the number of spines in cortical areas. Interestingly, the reduction is not homogenous, affecting for the most part the small spine population. In that respect, the mean volume of “thin” spines directly correlates with better skills, whereas larger volumes are predictive of slower learning (Dumitriu et al., 2010), supporting a relationship between spine size and learning.

Perhaps the most direct evidence linking spines and learning comes from zebra finches. In these birds, *in vivo* imaging studies have revealed that hearing loss decreases the size and stability of spines on neurons that provide input to the striatothalamic pathway, an area important for audition-dependent plasticity (Tschida and Mooney, 2012). In these species, song tutoring leads to a high spine turnover, while isolated birds present a lower turnover (Roberts et al., 2010). Thus, the key process in the bird’s brain is not only the ratio between small and large spines, but also the ability to modify the spinogenesis rate.

Our group has previously demonstrated that activation of the PI3K–Akt–GSK3 pathway increases synaptogenesis and spinogenesis and improves learning in mammals and invertebrates (Acebes and Morales, 2012). In this study, we present a quantitative analysis showing that PI3K activation improves a contextual learning process and specifically favors the formation of small, “thin” spines in both rat hippocampus and cell culture. Furthermore, our data indicate that PI3K spine formation is independent of neuronal activity; it relies on NMDA receptor activation and correlates with an upregulation of glutamic receptor expression. Finally, we have proved that PI3K activation is also able to induce the formation of new spines in a transgenic model of Alzheimer’s disease.

Our findings point to a connection among PI3K activation, small spine growth, and a structural correlate of learning, indicating that PI3K plays a role in synaptic structural plasticity.

## MATERIALS AND METHODS

### ANIMALS

Animal care procedures were conducted in accordance with the guidelines set by European Community Council Directive 86/609/EEC, and were approved by the Ethical Committee of the CIBIR and the Cantonal Veterinary Authorities (Vaud, Switzerland).

### BEHAVIORAL EXPERIMENTS

The protocol used was described by Cuesto et al. (2011). A total of 46 male adult Sprague Dawley rats (Harlan Laboratories), weighing 250–275 g, were individually housed in cages and allowed to acclimate for 1 week. 12-h light/dark cycle and temperature (20°C) conditions were controlled. Food and water were provided without restriction. Rats were handled daily for 2 min for 1 week just before the behavioral tests. After the acclimation period a stainless steel guide cannula was implanted aimed at the right dorsal hippocampus. The animals were anesthetized with xylazine (10 mg/kg) and ketamine (70–100 mg/kg), and mounted in a stereotaxic apparatus. A small hole was drilled through the skull, bregma coordinates: -2.8 mm anteroposterior, -1.5 mm lateral and -2.8 mm dorsoventral (Paxinos and Watson, 1998). The cannula was fixed to the skull using dental acrylic glue (Duralay 2244, Reliance). After surgery, the animals were allowed to recover for at least 5 days before performing the behavioral tests. All rats received a total volume of 1 μl per injection. PTD4 or PTD4-PI3KAc (20 μg/μl) was diluted in CSF to the final volume.

### ELEVATED PLUS MAZE

Since it is known that stress impairs spatial memory (Sandi et al., 2005), in order to reduce variability between groups, animals were subjected to an elevated plus maze (EPM) protocol to assign them to groups of equivalent anxiety levels, as previously described (Cuesto et al., 2011). The behavior of each rat was monitored using a video camera, and was recorded and analyzed with the aid of a computerized tracking system (Ethovision 3.0, Noldus Information Technology). The time elapsed before entering an open arm was found to be proportional to the anxiety level of the animal, as described by Herrero et al. (2006).

### CONTEXTUAL FEAR CONDITIONING

Contextual fear conditioning (CFC) is a hippocampus-dependent contextual related memory process (Kim and Fanselow, 1992; Phillips and LeDoux, 1992; Lopez-Fernandez et al., 2007). Twenty-four hours after injection animals were subjected to a training session. Both training and testing took place in two identical rodent observation cages placed inside a sound-attenuating chamber (Panlab), as described by Cuesto et al. (2011). During the training, the animal was allowed to explore the chamber freely for 160 s, after which time it received three electrical foot shocks (0.5 mA, 1 s). Thirty seconds later, the animals were removed and placed in their home cages. Testing was performed 1 day later, exposing the animal for 6 min to the same training context, and scoring freezing time as an index of fear. Freezing was defined as behavioral immobility (except for respiration movements) for at least 2 s. The context discrimination test took place on day three, employing a novel chamber with a different contextual background. The animal’s behavior was video recorded and later scored with the help of a video tracking system (Ethovision 3.0). To perform statistical analyses, freezing times were transformed into freezing percentage values. A control group of**naive animals remained in their cages and were subjected to the same treatment, injection and handling procedures except for the behavioral tests.

### PI3K-ACTIVATING PEPTIDES

Regulated activation of PI3K was achieved by using a peptide named PTD4-PI3KAc (Cuesto et al., 2011), which consists of a transduction domain, PTD4 (Tyr–Ala–Arg–Ala–Ala–Ala–Arg–Gln–Ala–Arg–Ala; Ho et al., 2001), fused to a phosphopeptide containing the intracellular phosphorylated domain of the PDGF receptor (Gly–Ser–Asp–Gly–Gly–pTyr–Met–Asp–Met–Ser; Derossi et al., 1998). This peptide has been shown to induce PI3K activation both in culture and *in vivo* brains (Cuesto et al., 2011). As control, we used the transduction domain PTD4 without the PI3K interaction domain of the peptide. Peptides were purchased either from GenScript, USA or the PolyPeptide Group, France.

### GOLGI STAINING AND SPINE DENSITY QUANTIFICATION

Animals were perfused transcardially with saline and 4% phosphate buffered paraformaldehyde. The injected hemispheres were processed for Golgi impregnation following the instructions of the FD Rapid GolgiStain^TM^ Kit (FD NeuroTechnologies, Inc., USA). Coronal sections obtained in a vibratome (100 μm thick) were mounted on gelatinized slides and coverslipped with distyrene dissolved in toluene–xylene (DPX; Sigma) mounting medium (the number of studied animals is shown in **Table 1**). CA1 basal dendrites from the dorsal hippocampus were selected for the analysis of spine density. Only lateral spines were measured, ignoring spines located on the top or bottom surface of the dendrites. Therefore, we assume that the results obtained were presumably an underestimate of the total number of spines. For the analysis, we selected dendritic segments starting 45 μm away from the cell body, as spine density reaches a constant value (Ruiz-Marcos and Valverde, 1969; Elston and DeFelipe, 2002). Images were taken using a Confocal SP5 Microscope (Leica) with bright field/transmission option, a 63× oil immersion lens. Pictures were deconvoluted employing the Acoloma macro (plugin for ImageJ; version 1.47, NIH, USA). Spine density was measured manually in the stacks using the ImageJ Plugin Cell Counter. Spines were marked in the appropriate focal plane preventing any double counting of spines. Maximum projections were used to measure dendrites length.

**Table 1:** Quantification of spine density *in vivo.*

Animal type	Number of animals	Number of dendrites	Total number of spines
**Naive**
Control 72 h	5	40	2527
PI3K 72 h	4	37	3120
Control 96 h	2	30	2439
PI3K 96 h	2	31	2225
**CFC**
Control 96 h	3	46	3832
PI3K 96 h	4	62	5044

*Summary data from naive and CFC animals selected for the study of spine density.
*

### SPINE MORPHOLOGY ANALYSIS

Spines were first categorized as spines with or without a clear neck (stubby spines). Measurements of the head area were manually and blindly performed using the ImageJ software and an adaptation of the protocol described by Verkuyl and Matus (2006). Briefly, a single image from the stack was selected for analysis and the scale was set. Each spine was selected, duplicated, rescaled (*X* and *Y* scales were set at 25), and thresholded until only the spine was selected. The “Polygon selections” tool was used to define the perimeter of the head, splitting it from the neck or the dendritic shaft. Area measurements were obtained from this image. Number of spines analyzed is shown in **Table 2**.

**Table 2:** Analysis of spine morphology *in vivo.*

Animal type	Number of animals	Number of dendrites	Total number of spines
**Naive**
Control (72 h + 96 h)	7	26	819
PI3K 72 h	5	17	697
PI3K 96 h	2	8	202
**CFC**
Control 96 h	3	16	300
PI3K 96 h	4	18	413

*Summary data regarding the analysis of spine morphology of naive and CFC animals.*

### *IN VIVO* ELECTROPHYSIOLOGY AND PHARMACOLOGY

Under isoflurane anesthesia, three Sprague Dawley male rats (500–700 g) were implanted with 32-site linear multisite silicon probes (Neuronexus, Ann Arbor, MI, USA) for chronic recording. Sites were spaced 50 μm apart and spanned the CA1 and CA3/DG regions, as guided by the characteristic evoked potentials elicited through a stimulating electrode placed in the ipsilateral CA3, and which was removed after implantation. In addition, a plastic guide cannula (Micro-Line, 7.6 mm i.d.) was placed above the right lateral ventricle: anteroposterior: -1 mm; lateral: 1.5 mm (Paxinos and Watson, 1998) and fixed with dental acrylic glue. Two micro-screws were drilled into the skull for anchoring purposes and as a reference for recording. Following a 1-week recovery period, plus three additional days for control recordings, the animals were anesthetized again with isoflurane, and 1 μl (20 μg) of PTD4-PI3KAc was slowly (1 min) injected into the ventricle through the cannula of a Hamilton syringe. Fifteen minutes later, the syringe was removed and the anesthesia was discontinued. Local field potentials (LFPs) were recorded with a wireless headstage (TBSI) at a sampling rate of 20 kHz, using MultiChannel System hardware and software (Reutlingen), while the animal was in its home cage awake and immobile.

### LOCAL FIELD POTENTIAL ANALYSIS

Depth profiles of ongoing LFPs were analyzed through independent component analysis (ICA), as previously described (Korovaichuk et al., 2010; Makarov et al., 2010). The ICA is a blind source separation technique suitable for spatial discrimination of mixed components fixed in space, thus, the ICA disentangles spatial maps of LFPs into the independent pathway-specific sources or LFP generators, based on their distinct spatial distribution. LFP generators represent time-envelopes of the compound synaptic activity elicited by specific and homogeneous afferent population (Fernández-Ruiz et al., 2012a,b; Benito et al., 2013), and each is described by a characteristic spatial distribution that is fixed in all animals and for all functional states, and a temporal evolution that is specific for the period analyzed. The mathematical validation and the interpretation of ICA components in laminated structures were performed using realistic LFP modeling (Makarova et al., 2011). To perform the ICA, we employed the KDICA algorithm (Chen, 2006). We used pre-processing of LFPs by principal component analysis that allows reducing the presence of remote generators and stabilizes the subsequent convergence of ICA in true stable LFP generators (Makarova et al., 2011). Cellular identity and chemical nature of LFP generators, as well as their stability across animals were previously assessed (Korovaichuk et al., 2010; Fernández-Ruiz et al., 2012a; Benito et al., 2013). Once extracted, virtual LFPs can be reconstructed for each generator by multiplying their spatial weights by the specific time course, thus regaining true absolute amplitude and polarity. We considered statistically significant LFP generators to be those contributing to at least 3% of the total LFP variance in the LFP profile. However, it should be stated that weaker generators are routinely encountered, which require further exploration to ensure their stability and quantitative reliability.

The evolution of the power of an LFP generator over time is described (in mV^2^) by the following equation: $P(t)=\int \;H\left(t-\tau \right)\nu^{2}\left(\tau \right)dt$, where *v(t)* is the virtual LFP at the electrode with maximal power and *H* is the appropriately scaled square kernel of the length. To minimize state-dependent bias, epochs were only analyzed while the animal was awake, quiet, and immobile in the cage. All recordings were obtained at a similar time of the day (between 15:00 and 18:00).

### HIPPOCAMPAL CELL CULTURES

Primary neuronal cultures were prepared from postnatal hippocampi (P0–P1 rat pups), as previously described (Cuesto et al., 2011). Briefly, glass coverslips were coated with poly-L-lysine (100 μg/ml) and laminin (4 μg/ml). Neurons were seeded at 15 × 10^4^/cm^2^ and grown in a culture medium consisting of neurobasal medium (Invitrogen), supplemented with glutamine 0.5 mM, 50 mg/ml penicillin, 50 U/ml streptomycin, 4% FBS, and 4% B27 supplement (Invitrogen). After 4, 7, 14, and 21 days in culture, 100 μl (of a total of 500 μl) of culture medium was replaced by 120 μl of fresh medium. In the case of neurons cultured in glass bottom microwell dishes (MatTek corporation), 400 μl (of a total of 2000 μl) of culture medium were replaced by 500 μl of fresh medium. On day 4, cytosine-D-arabinofuranoside (4 μM) was added to prevent overgrowth of glial cells. Hippocampal cultures were routinely treated for 48 h, starting at 19 days *in vitro* (DIV). Controls were sister cultures grown in the same multiwell plate. The PI3K inhibitor LY294002 was from Sigma. TTX and dl-APV both from TOCRIS, were administered at day 19DIV immediately after induction with PTD4 or PTD4-PI3KAc.

### TRANSFECTION

In order to visualize their spines in culture, neurons were transfected with a plasmid encoding the green fluorescent protein (GFP), fused with chick β-actin under the control of the platelet-derived growth factor promoter region (kindly provided by Y. Goda, MRC Cell Biology Unit, University College London, London, UK; Colicos et al., 2001). Electroporation was performed before plating using a BioRad Cell electroporator system. Approximately 4 × 10^6^ cells and 10 μg of plasmid were mixed in BioRad electroporation buffer (BioRad). An exponential discharge protocol with the following parameters was employed: 220 V, 950 μF and resistance fixed to infinitum.

### RNA EXTRACTION, REVERSE TRANSCRIPTION, AND REAL-TIME RT-PCR

For PCR analysis, total RNA was extracted using the Tripure^TM^ Isolation Reagent (Roche), according to the manufacturer’s instructions. The recovery of RNA was similar between the different assay conditions. Reverse transcription was performed using random hexamer primers, exactly as previously described (Gavilán et al., 2009). cDNAs were diluted in sterile water and used as template for amplification by polymerase chain reaction. Optimization and amplification of each specific gene product was performed using the ABI Prism 7000 sequence detector (Applied Biosystems) and TaqMan probes designed by Applied Biosystems, as described in Gavilán et al. (2013). cDNA levels were determined using two different housekeepers (GAPDH and β-actin). The amplification of the housekeepers was done in parallel with the gene to be analyzed. Similar results were obtained with both housekeepers. Results were normalized using both β-actin and GAPDH expression. Threshold cycle (Ct) values were calculated using the software supplied by Applied Biosystems.

### IMMUNOCYTOCHEMISTRY AND IMAGE ANALYSIS OF SPINES IN CULTURES

To analyze spine density, cultures of actin-GFP transfected neurons were fixed and processed as described by Cuesto et al. (2011). Cultures were rinsed in PBS and fixed for 10 min in 4% paraformaldehyde/PBS. A polyclonal antibody against Synapsin (ref. 2312, Cell Signaling) was employed to quantify presynaptic contacts. Coverslips were incubated overnight at 4°C, washed three times in PBS and incubated for 30 min in PBS solution containing the fluorescence-conjugated secondary antibody (Alexa Fluor 555, Invitrogen), washed and mounted in Mowiol. To reduce variability among different cultures and treatments, spines were exclusively analyzed in proximal dendrites, starting from a clearly identified neuronal cell soma. Pictures were obtained with a Leica Confocal SP5 Microscope, and individual images were acquired in 1024 × 1024 stack pictures (pixel size between 90 and 60 nm with a 0.5 μm *z*-step). Spine density and dendritic length were calculated manually with ImageJ.

### TIME-LAPSE ANALYSIS

For time-lapse imaging cultures plated in glass bottom microwell dishes (MatTek Corporation) were mounted in a temperature and atmosphere controlled chamber (35–37°C and 5% CO). Images were taken using a Confocal SP5 Microscope (Leica) with a 63× oil immersion lens. The laser power was set at 5–8% to minimize phototoxicity. Image resolution was 1024 × 1024, with a pixel size between 90 and 60 nm. Stack images (1 μm *z*-step) were taken every 2 min for a total of 10 min. Live images of the same dendrites were initially obtained at 19DIV and after 48 h (21DIV). PTD4 or PTD4-PI3KAc was administered after the 19DIV visualization. The Leica SP5 mark and find module was used to search for the same neuron on different days. To account for miscalculation due to spine motility among *z*-planes, spine density was quantified in the first and the last stacks of each recording, and the value obtained was the mean of both values. Maximum projection pictures were deconvoluted using the Acoloma plugin (ImageJ) prior to the analysis. Differences due to spine loss or spine formation were expressed as percentage of change. The spine head area study was performed using ImageJ as previously described for the brain sections.

### TRANSGENIC MICE COLONY AND STEREOTAXIS

Tg2576 (B6;SJL:-Tg(APPSWE)2576) Alzheimer’s disease transgenic mice were obtained from Taconic (USA, ref. 1349). These animals express the human 695-aa isoform of the amyloid precursor protein (APP) containing the Swedish double mutation (APPswe), driven by a hamster prion promoter (Hsiao et al., 1996). The colony was established by mating heterozygous males with B6SJLF1 female mice and genotyped with the primers: 1503: CTGACCACTCGACCAGGTTCTGGGT and 1502: GTGGATAACCCCTCCCCCAGCCTAGACCA from Taconic. Stainless steel guide cannulas were implanted in the mice, aimed at the lateral right ventricle. The animals were anesthetized with isoflurane and mounted in a stereotaxic apparatus. A cannula was fixed through a hole drilled at the skull (bregma coordinates: -0.6 mm anteroposterior, -1.2 mm lateral, and -1.7 mm dorsoventral; Paxinos and Franklin, 2001). After the surgery, animals were allowed to recover for at least 5 days before injection. A final volume of 4 μl, containing 20 μg of PTD4-PI3KAc, was injected. PTD4-FITC was used as a control.

### BIOLISTIC LABELING

To quantify spine density in Tg2576 transgenic mice, a Biolistic (DiI) protocol was employed. Neuron labeling was performed using the Helios Gene Gun System (BioRad), following the protocol described in Grutzendler et al. (2003). Briefly, a suspension containing 3 mg of DiI or DiO (Molecular Probes, Invitrogen) dissolved in 100 μl of methylene chloride (Sigma) and mixed with 50 mg of tungsten particles (1.7 μm diameter, BioRad) was spread on a glass slide and air-dried. The mixture was resuspended in 3.5 ml distilled water and sonicated. Subsequently, the mixture was drawn into a Tefzel tubing (BioRad); the tubing was then dried under a nitrogen flow gas and cut as bio gun cartridges (13 mm long). Particles were delivered to the hippocampus using a modification of the gun to enhance accuracy by restricting the target area (O’Brien et al., 2001). To obtain a double stain, in some slices sequential shooting of both DiI and DiO bullets were used. Shooting was performed over 200 μm vibratome coronal sections at 80 psi through a membrane filter (Millipore, ref. TSTP04700). Sections were stored protected from light at room temperature in PBS for 3 h, incubated with DAPI and mounted in Mowiol prior to visualization. DiI-labeled pyramidal neurons from CA1 of the dorsal hippocampus were imaged using a Leica confocal SP5 with a 63× oil-immersion objective (optical zoom 1×). Spine density was analyzed along the primary basal dendrites using a Sholl analysis, with a similar criterion as that described in the Golgi staining section.

### STATISTICS

All data are represented as means ± SEM and were analyzed using Prism software (version 4.0, GraphPad). Statistical analysis was performed using a Student’s *t*-test as appropriate. A Kolmogorov–Smirnov test was used for the analysis of dendritic spine areas. A two-way ANOVA test was used to compare spine densities in the Tg2576 model. Significant levels were noted as follows: *p < 0.05, **p < 0.01, and ***p < 0.001. All statistics are included in the figure legends.

## RESULTS

### PI3K OVERACTIVATION IMPROVES LEARNING ABILITIES IN RATS

**FIGURE 1:**
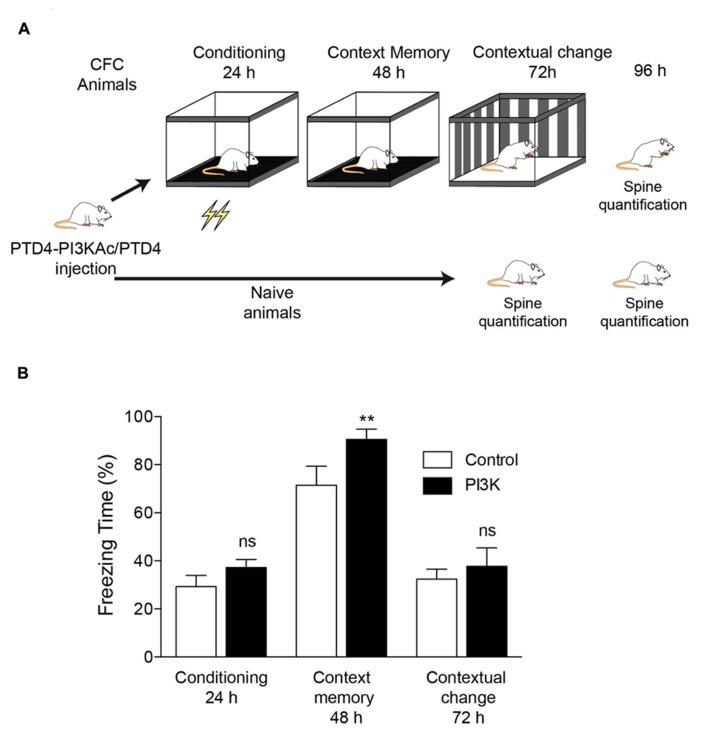
**PI3K activation improves a hippocampal-dependent learning behavior. (A)** Illustration depicting the experimental design. All animals were subjected to the same handling treatment before the experiments. At time 0, they were injected either PTD4-PI3KAc or PTD4 as control. The experimental group was subjected to the CFC test (CFC animals), whereas the control group (naive animals) remained in their cages throughout the entire experiment. **(B)** CFC test results for animals injected with either PTD4-PI3KAc (black bars, *n* = 10) or the PTD4 control transduction domain (open bars, *n* = 10). No differences in freezing were observed during conditioning (24 h), indicating normal fear acquisition and no differences in sensitivity to the shocks. In the context memory test (48 h), PTD4-PI3KAc injected rats showed higher freezing levels than control rats when re-exposed to the conditioning chambers with no shock delivery. A contextual change to discard unspecific effects (72 h) showed no differences among animals, indicating that the memory effects are context-dependent (Student’s *t*-test). Animals were sacrificed 24 h later, i.e., 96 h after peptide injection.

To investigate structural correlates of hippocampus-dependent learning, we used the CFC paradigm. Animals were distributed into two categories, naive and CFC. Previous to the experiment, all animals were surgically implanted a steel cannula at the hippocampus dorsal area (**Figure 1A**). PTD4-PI3KAc was injected into the dorsal hippocampus to investigate behavioral changes specific to this brain area (**Figure 1A**).

CFC animals were divided into two equivalent groups of equivalent anxiety levels; one group was injected with the PTD4 control transduction domain (CFC control condition; *n* = 10) and the second with PTD4-PI3KAc (10 μg; *n* = 10). The naive group was similarly injected with either PTD4-PI3KAc (treatment) or PTD4 (naive control animals; **Figure 1A**). Twenty-four hours after injection, the animals were subjected to a CFC test. The results showed improved learning abilities in PTD4-PI3KAc-injected animals, i.e., they spent more time in a frozen state than the control animals, a manifestation of higher conditioned fear in response to the context (**Figure 1B**). This freezing time increase in PTD4-PI3KAc-treated rats was not due to non-specific changes in generalized fear or anxiety, as evidenced by the fact that their freezing levels were similar to those of the controls when exposed to a novel context test (i.e., contextual change; **Figure 1B**).

### SPINE DENSITY IN THE HIPPOCAMPUS IS UPREGULATED BY CFC AND PI3K ACTIVATION

**FIGURE 2:**
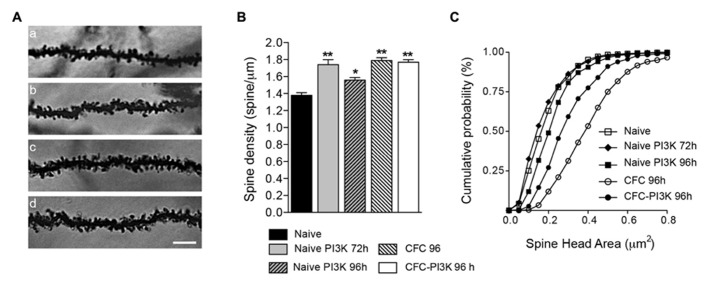
**PI3K activation and CFC modified spine density and head area. (A)** Representative images (96 h after injection) of stratum oriens dendrites in the CA1 region of the hippocampus from Naive (a), Naive injected with PTD4-PI3KAc (b), CFC (c), and CFC injected with PTD4-PI3KAc (d) animals. Scale bar = 5 μm. **(B)** Mean average graph of spine density changes during: naive conditions (1.38 ± 0.03 spines/μm), after 72 and 96 h of PI3K activation (1.74 ± 0.06 and 1.56 ± 0.03 spines/μm, respectively) and after the CFC test (CFC-96 h; 1.79 ± 0.02 spines/μm and CFC-PI3K-96 h: 1.77 ± 0.03 spines/μm) (Student‘s *t*-test). **(C)** Cumulative frequency distribution of spine head areas under the experimental conditions. In naive animals, no effect was seen after 72 h, but a significant increased in spine head area was found 96 h after PI3K activation (Kolmogorov–Smirnov, *p* < 0.001), whereas CFC alone induced an even more pronounced shift toward large spine areas. Interestingly, in CFC tested animals, PI3K overactivation reduced spine head areas as compared to the CFC controls (Kolmogorov–Smirnov, *p* < 0.001).

To describe changes in spine distribution, brains were then processed for Golgi staining impregnation, and basal dendrites from pyramidal neurons of CA1 were analyzed to quantify spine density (**Table 1**), comparing CFC and naive animals (**Figure 1A**). Brains from CFC animals were fixed 24 h after finishing the test (i.e., 96 h after injection), while brains from naive animals were fixed 72 and 96 h after injection. Analysis of the naive cohort reveals that PTD4-PI3KAc increased spine density by 26% after 72 h of injection, albeit the increase after 96 h amounted to only 13% (statistically different; **Figure 2B**). This time-dependent reduction might reflect the activity life of the PI3K activator peptide *in vivo* (**Figure 2B**). Interestingly, the CFC by itself increased spine density around a 29% when compared with the naive animals, while the PTD4-PI3KAc-injected animals showed no differences in spine density as compared to CFC animals (1.77 ± 0.03 versus 1.79 ± 0.03 spines/μm in PI3K and CFC animals, respectively; **Figure 2B**). This would indicate that the learning behavior associated with the CFC test induced a reorganization of spine density that overcame the observed PI3K effects; however, animals injected with PTD4-PI3KAc exhibited better cognitive abilities (**Figure 1B**).

### PI3K FAVORS THE FORMATION OF SMALL HEAD SPINES

Despite the superior contextual fear memory retention observed in PTD4-PI3KAc-treated animals as compared to vehicle-treated rats, these groups did not differ in hippocampal CA1 spine density. This raised the question as to whether structural changes in spines could be responsible for the behavioral differences observed. To this end, we performed a quantification of spine head areas in the different experimental groups.

Spines undergo morphological changes related to the strength of their synaptic contacts. In this regard, they have been conventionally classified as stubby, thin and mushroom-shaped (Peters and Kaiserman-Abramof, 1970; Harris et al., 1992). Such classification depicts a static picture of a dynamic process. For a more accurate description of the shape changes, we have studied individual spine head areas as a continuous distribution (Dumitriu et al., 2010; Peebles et al., 2010). The distribution yielded a compelling result (**Figure 2C**): after 72 h of PTD4-PI3KAc injection, even though spine density increased a 26%, head areas did not show any significant change, and only after 96 h a slightly change in head areas was noticeable (closed squares in **Figure 2C**). In clear contrast, spines of CFC animals showed a shift toward higher area values, statistically significant, having the largest mean values of all groups (open circles in **Figure 2C**). Interestingly, CFC animals injected with PTD4-PI3KAc (closed circles) exhibited a population of spines that were smaller in size as compared to control CFC animals (open circles; **Figure 2C**).

**FIGURE 3:**
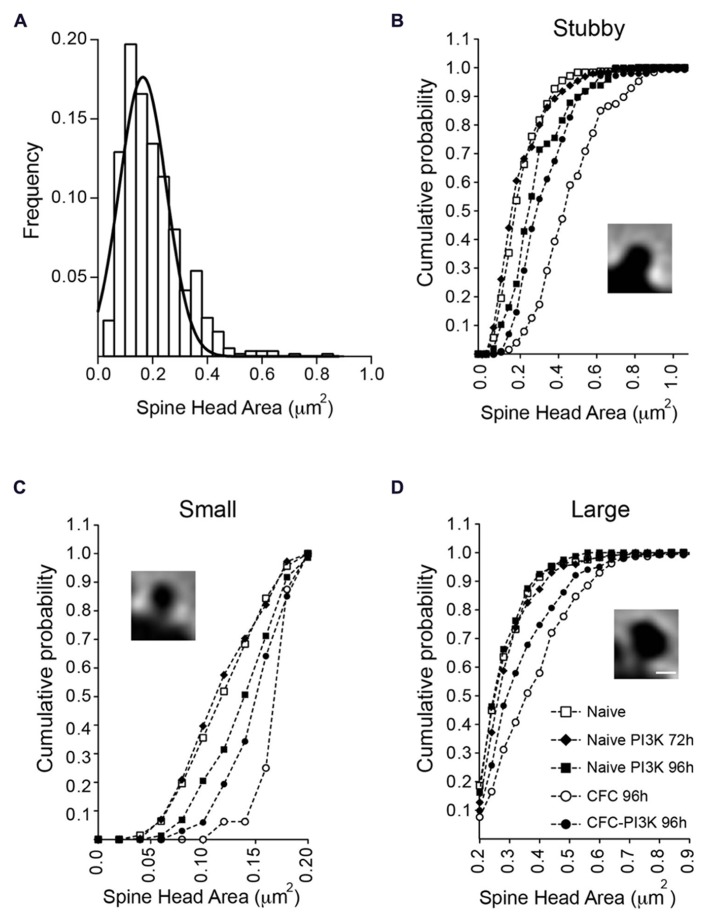
**Changes in spine head area are independent of spine morphology. (A)** Normal distribution of necked spine head areas from the naive reference group (72 and 96 h combined). No-neck spines (stubby) were not included in this distribution. The mean spine head area of the distribution (0.19 ± 0.004 μm^2^) was used to further subdivide necked spines into small (below the mean) and large (above the mean) area spines. **(B–D)** Head area cumulative frequency plots for the different morphological spine types and experimental conditions. Naive animals injected with PTD4-PI3KAc exhibited a statistically significant shift toward larger areas after 96 h (closed squares), but only in the small and stubby categories (Kolmogorov–Smirnov, stubby: *p* < 0.001, small: *p* < 0.01). CFC animals showed significantly larger head sizes in all three spine categories (open circles; Kolmogorov–Smirnov, *p* < 0.001), whereas activation of PI3K (closed circles) in CFC tested animals favored a shift toward small spine heads, as compared to CFC alone (Kolmogorov–Smirnov, *p* < 0.001). Scale bar: 1 μm.

To determine whether there was a global change in spine head or it was rather specific for a particular subtype of spines, we analyzed the temporal distribution, dividing the spines into three categories (**Figures 3B–D**). To this end, we first classified all the spines identified as having no neck (stubby spines) or having a clear neck. For the latter, we next measured the head area of all the spines in the naive condition group at both 72 and 96 h. Since the results were very similar (0.21 ± 0.005 μm^2^ versus 0.22 ± 0.006 μm^2^ for 72 and 96 h, respectively), we grouped both conditions into a single control group and performed a distribution analysis of the total head areas population, the data was fit to a normal Gaussian distribution with a mean value of 0.199 ± 0.004 μm^2^ (Gaussian fit *r*^2 ^= 0.91; **Figure 3A**). Based on this estimated value, the spine population was arbitrarily divided into small spines, i.e., those with areas below the mean size (head areas < 0.199 μm^2^); large spines with areas larger than the mean value (head areas > 0.199 μm^2^), and a third category including all the non-neck spines (stubby spines).

The individual analysis of the three categories indicated that in naive conditions regardless of the spine type, PI3K activation failed to induce morphological changes at 72 h (**Figure 3**; **Table 3**). After 96 h, however, PI3K activation promotes a remodeling of spines toward broad areas on both the stubby and the small spine populations. The large spine population, on the other hand, was very stable, showing no significant changes during the first 96 h (**Figure 3**; **Table 3**). The CFC test by itself, induced an evident shift toward larger areas in all types of spines, with a more obvious change in the category of small spines (0.126 ± 0.002 versus 0.171 ± 0.005; **Figure 3**; **Table 3**). Importantly, CFC animals treated with PTD4-PI3KAc showed reduced spine head areas in all three categories compared to CFC controls (**Figures 3C,D**; **Table 3**). Altogether, the morphometric analyses indicate that CFC test induce a remodeling of spine size, while PI3K activation induces a structural remodeling that favors the formation of small head spines. Thus, even in the brains of animals subjected to the fear conditioning test, PI3K kept the morphology of the spines in a thin-like phenotype category.

**Table 3:** Mean spine head area (μm^2^).

Conditions/spines	Stubbies	Small head	Large head
**Naive**
Controls (72 h + 96 h)	0.239 ± 0.008	0.126 ± 0.002	0.296 ± 0.006
PI3K 72 h	0.236 ± 0.010	0.125 ± 0.002	0.307 ± 0.007
PI3K 96 h	0.320 ± 0.022	0.149 ± 0.006	0.292 ± 0.009
**CFC**
Control 96 h	0.494 ± 0.016	0.171 ± 0.005	0.397 ± 0.011
PI3K 96 h	0.362 ± 0.013	0.157 ± 0.003	0.349 ± 0.009

*Summary data of the mean size area of each spine category. Spine areas values in control-naive conditions, after 72 and 96 h were pooled together because there was not statistical difference between them. Control animals in CFC conditions are animals that underwent the CFC test but were injected with the peptide control*

### *IN VIVO* PI3K ACTIVATION INDUCES CHANGES IN SCHAFFER-CA1 INPUT

**FIGURE 4:**
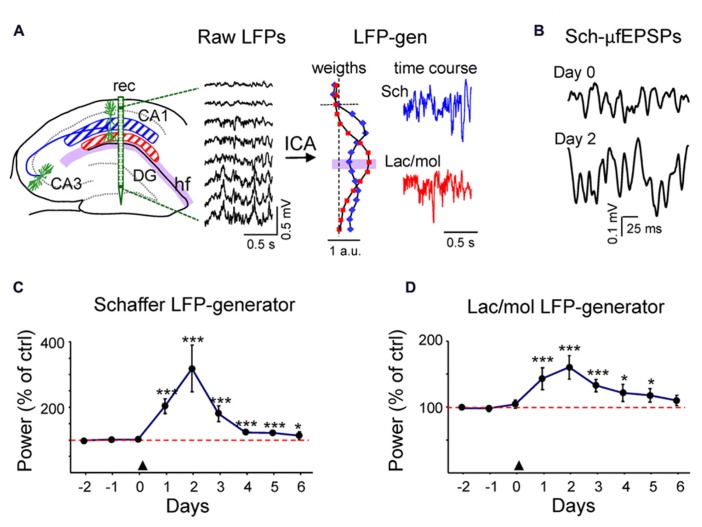
***In vivo* transient increment of ongoing activity in synaptic inputs to CA1. (A)** Hippocampal raw LFPs recorded in depth profiles by linear probes (rec in left scheme). Only every other site is represented. The ICA analysis of linear raw LFPs rendered two main LFP generators in the CA1 region, one centered in the striatum radiatum or Schaffer-LFP generator (blue) and other in the striatum lacunosum-moleculare (lac/mol generator; in red). Each had a characteristic spatial distribution (see normalized relative spatial weight in vertical plots), while the temporal activation was specific to the epoch analyzed (color traces to the right). The baseline activity of the Schaffer LFP generator consists of a regular succession of small field potential wavelets at gamma frequency termed μ-fEPSPs [enlargement in **(B)**]. **(C,D)** LFP activity was recorded during the three days prior to PTD4-PI3KAc injection (marked by an arrowhead at day zero) and for 6 days afterwards. The power of an LFP generator estimates the magnitude of the ongoing synaptic input from a specific afferent pathway to the target population (CA1 pyramids). It was calculated in 200-s epochs and plotted for each day as a percentage of the mean power of the control (red dashed line), estimated as the average of the 3 days before injection). Both the Schaffer **(C)** and the lac.-mol. **(D)** LFP generators exhibited a strong power increment that peaked 48 h after the treatment and decayed over the following days (Student’s *t*-test). Sample traces of reconstructed virtual LFPs at Day 0 (control) and two days post-injection are shown in **(B)**.

To explore whether changes in spine density as the result of PI3K activation were functional in intact animals, we quantified the ongoing synaptic activity in chronic recordings using pathway-specific LFP generators extracted from laminar LFP-profiles across the CA1/DG (Benito et al., 2013; Fernández-Ruiz et al., 2013). As in previous studies, we found two dominant generators in the CA1 region that displayed maximum activity in the middle and the distal apical dendrites, respectively. The first corresponded to a well-characterized ipsilateral CA3 input to the CA1 region (the Schaffer LFP generator, blue plot and trace in **Figure 4A**; Fernández-Ruiz et al., 2012a), and the other to an as yet unidentified mixture of synaptic inputs to the stratum lacunosum-moleculare (Lac-mol generator, red plot, and trace). The active synaptic zones, determined in Benito et al. (2013), are centered at the maximum peaks of the corresponding bell-shaped distributions of spatial weights in **Figure 4A**. Both signals were highly consistent throughout the dorsal hippocampus, thus ensuring the stability of measurements in inter-animal comparisons.

Following reconstruction of generator-specific (virtual) LFPs, the baseline activity of the Schaffer LFP generator consisted of a rather regular succession of small (40–200 μV) negative field potential waves at gamma frequency (**Figures 4A,B**). These waves correspond to excitatory packages elicited in the CA1 pyramidal population by the synchronous firing of CA3 assemblies, the so-called micro-field excitatory postsynaptic potentials (μ-fEPSPs; Fernández-Ruiz et al., 2012a). The baseline activity in the lac/mol LFP generator was more variable in the frequency domain, and displayed slow and fast waves of both polarities. When periods of LFP (200-s long) were recorded during an awake/alert state over successive days, we found a marked increase in the mean variance of the two LFP generators beginning 24 h after ventricular injection of PTD4-PI3KAc. The increased spontaneous activity reached a maximum value at day 2 (**Figures 4C,D**) (319 ± 78% and 160 ± 18% of the pretreatment value for the Schaffer and lac/mol LFP generators, respectively, *n* = 3) and decayed thereafter, albeit with faster rate in the Schaffer LFP generator. The pathway-specificity of the Schaffer generator allowed the quantification of individual gamma waves or μ-fEPSPs. These were sorted and their amplitude and duration distributions were estimated. Data were pooled in three days periods, pre-treatment (control), post-injection (days 1–3 post-treatment) and recovery (days 4–6). There was no variation in the mean and maximum duration of μ-fEPSPs or the mean rate following treatment, while a stable, significant increase of the mean and maximum amplitude values of the μ-fEPSPs distributions (176 ± 15% and 170 ± 6%) were observed post-injection and during the recovery period (111 ± 10% and 126 ± 16%). Since the parameters related to the time dynamics of the afferent CA3 assemblies did not change (presynaptic frequency and synchronization are responsible for the postsynaptic rate and duration of μ-fEPSPs, respectively), the amplitude changes were most likely due to increased unitary synaptic currents in the postsynaptic CA1 domain, which is consistent with the observed increase in spine density.

### PI3K SPINE FORMATION IS INDEPENDENT OF NEURONAL ACTIVITY

To investigate the role of synaptic activity in the PI3K-induced spine formation process, we moved on to a more simple system, hippocampal neurons in culture. To this end, we used hippocampal neurons expressing actin-GFP (Colicos et al., 2001) of 3 weeks old and studied them from days 19 to 21 in culture. At this stage, neurons have reached a fully developed morphology, characterized by a close-knit network of dendrites decorated with a high density of spines.

**FIGURE 5:**
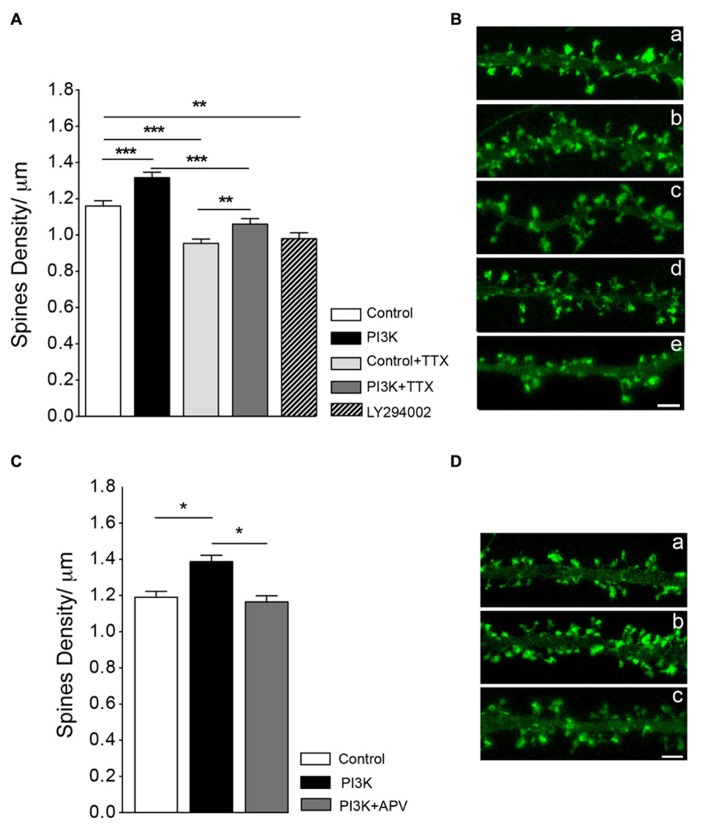
**Synaptic activity does not modulate spine formation in culture. (A)** Neuronal activity is not required for PI3K-induced spine density increase. Spine density was quantified in cultures with and without PTD4-PI3KAc under normal conditions (control versus PI3K) and in cultures treated with TTX (2 μM) for 48 h (control + TTX versus PI3K-TTX). Compared to the control cultures, PTD4-PI3KAc significantly increased spine density regardless neuronal activity (from 1.16 ± 0.03 to 1.32 ± 0.03 spines/μm without TTX and from 0.95 ± 0.02 to 1.06 ± 0.03 spines/μm with TTX). (Student’s *t*-test). Treatment with a PI3K inhibitor activity for 48 h, reduces spine density a 15% (0.9 ± 0.03 spines/μm). **(B)** Representative dendritic segments of actin-GFP transfected neurons after 21DIV in control (a), PTD4-PI3KAc-treated (b), TTX-treated (c), TTX + PTD4-PI3KAc-treated (d) and LY294002-treated cultures (e). Scale bar = 2.5 μm. The number of dendrites analyzed in the different cultures was: 95 for Control, 91 for PI3K, 109 for control + TTX, 113 for PI3K + TTX and 137 for LY294002. **(C)** NMDAR receptor activation appears to be necessary for the formation of PI3K-induced spines. As before, spine density in response to PI3K activation was quantified in sister cultures under normal conditions (control versus PI3K) and in the presence of the NMDA receptor inhibitor d-APV (PI3K + APV). Spine density was significantly higher in PI3K as compared to the control culture (1.39 ± 0.04 versus 1.19 ± 0.03 spines/μm, respectively), whereas in the PI3K + APV culture, spine density was reduced back to control levels (1.16 ± 0.03 spines/μm) (Student’s *t*-test). **(D)** Representative dendritic segments from control (a), PI3K (b) and PI3K + APV (c) cultures. Scale bar = 2.5 μm. A total of 79 control; 73 PTD4-PI3KAc and 73 PTD4-PI3Kac+d-APV dendrites from four different cultures were analyzed.

First, we studied the dependence on synaptic transmission by exposing cultures to the sodium channel blocker TTX, which inhibits neural activity. TTX (2 μM) treatment reduced spines density by around 18%, as compared to a sister culture (**Figure 5A**). When cultures were co-treated with PTD4-PI3KAc and TTX, spine density increased 11.2% over cultures treated with TTX alone (**Figure 5A**). This percentage of increase was not statistically different from that observed after PI3K activation in the absence of TTX (13.4%, with PTD4-PI3KAc at a concentration of 21 μM). Therefore, we concluded that overactivation of PI3K leads to the formation of spines independently of neuronal activity.

### SPINE STABILITY PARTIALLY DEPENDS ON PI3K ACTIVITY

Since PI3K activation induces spine formation, we next wonder if PI3K activity was required to maintain spine density. To answer this question, neuronal cultures were incubated with 5 μM of LY294002, a chemical inhibitor of PI3K during 48 h. After the treatment spine density was reduced a 15.5% from a 1.16 ± 0.03 spines/μm in control conditions to a 0.9 ± 0.03 spines/μm (**Figures 5A,B**). This result indicates that PI3K participates in the regulation of spine stability.

### ACTIVATION OF NMDA IS REQUIRED FOR THE PI3K SPINE FORMATION PROCESS

As activation of the NMDA receptor was demonstrated to be crucial in the initial steps of spine formation (Kwon and Sabatini, 2011), we next analyzed the role of NMDA signaling in PI3K-induced spine formation. To do this, we blocked NMDA receptor activity using the competitive inhibitor d-APV (at a concentration of 25 μM). After addition of d-APV, we observed a slight, but non-significant reduction of 5% in spine density, as compared to the control cultures (data not shown). Nevertheless, when NMDA signaling was impaired, the effect of PI3K on spine induction was inhibited (**Figures 5C,D**). Therefore, we concluded that NMDA activation is necessary for the mechanism of PI3K induction of spines.

### NEWBORN SPINES IN CULTURE ARE ASSOCIATED WITH PRESYNAPTIC TERMINALS

**FIGURE 6:**
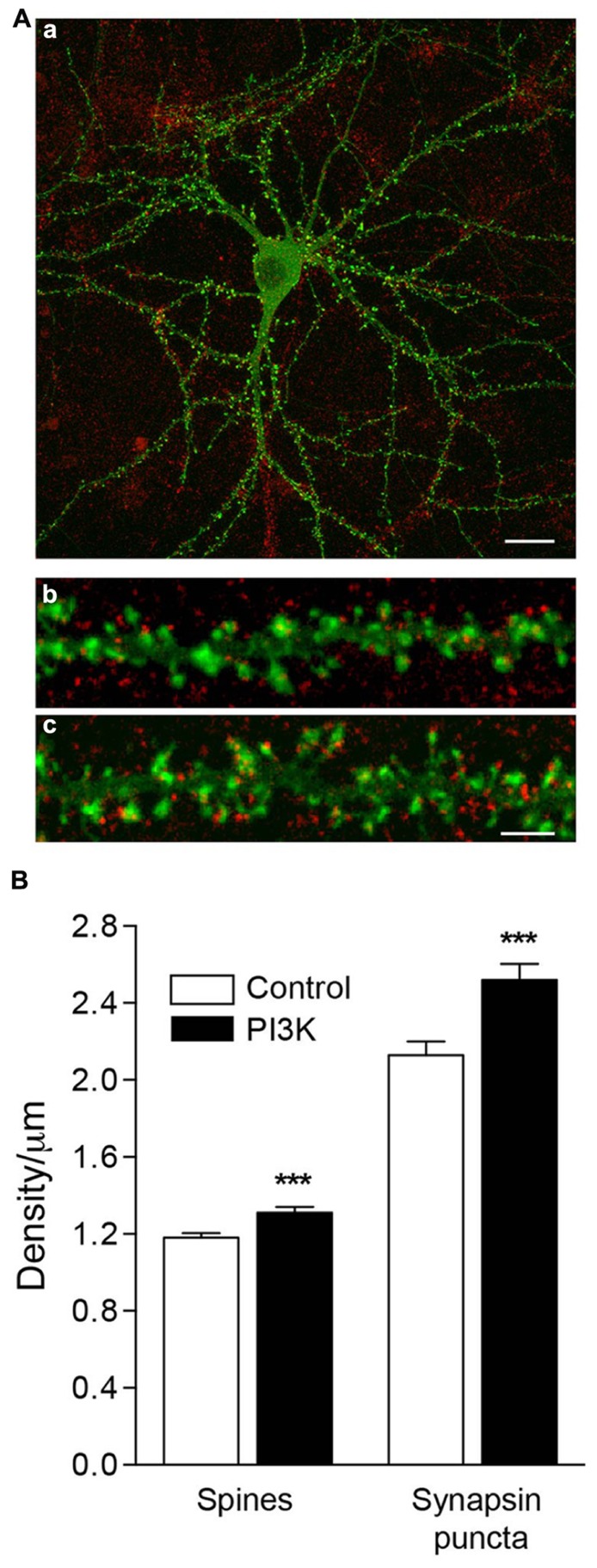
**PI3K activation induces the formation of functional spines in culture. (A)** An example of an actin-GFP (green) transfected neuron at 21DIV (a), fixed and immunostained for synapsin (red). Magnified images show a detailed section of dendritic spines under control conditions (b) and after 48 h of PTD4-PI3KAc addition (c). Synapsin puncta were clearly visible, associated with the spines. Scale bar = 20/2.5 μm. **(B)** Left bars: quantification of spine density after 48 h of PTD4-PI3KAc treatment. Control: 1.18 ± 0.02 and PTD4-PI3KAc: 1.31 ± 0.03 spines/μm. Right bars: quantification of synaptic puncta after 48 h of PTD4-PI3KAc treatment. Control: 2.13 ± 0.07 and PTD4-PI3KAc: 2.52 ± 0.08 synapse/μm. A total number of six individual cultures, 121 control and 118 dendrites from PTD4-PI3KAc neurons were used (Student’s *t*-test).

Our next question was whether synaptogenesis and spinogenesis are related processes, or on the contrary, they are independently regulated. To determine this, we analyzed the correlation between a presynaptic marker, synapsin, and the spine density in culture. Overactivation of PI3K increases the number of spines, raising their density by 11.2% (**Figures 6A,B**), from 1.18 ± 0.02 spines/μm in control cultures treated only with the PTD4 to 1.31 ± 0.03 spines/μm after the addition of PTD4-PI3KAc. In parallel, the density of synapsin puncta grew from 2.13 ± 0.07 to 2.52 ± 0.08 synapses/μm, an increase of 18.4% (**Figure 6B**). These data suggest that both processes might be regulated by different mechanisms.

In the same experiments, we quantified the average ratio of spines bearing synapsin puncta, and found that their proportion had increased significantly in the treated cultures, from 59 ± 1.4% in control cells to 67 ± 1.4% in PTD4-PI3KAc-treated cells (data not shown). Therefore, it can be concluded that PI3K activation increases the proportion of spines associated with a presynaptic terminal.

### PI3K ACTIVATION UPREGULATES GLUTAMATE RECEPTORS AND THE EARLY GENE ARC EXPRESSION

**FIGURE 7:**
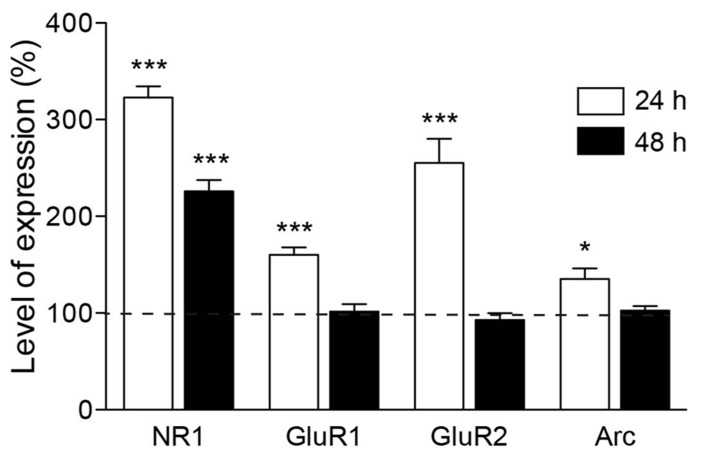
**Expression of glutamate receptor subunits is increased upon PI3K activation.** Time course of mRNA expression for different glutamate receptor subunits and Arc, after PTD4-PI3KAc treatment in culture. NMDA receptor NR1 subunits were upregulated after 24 h (white box) of peptide addition and decreased after 48 h (black box). Expression levels of GluR1, GluR2 and Arc showed a similar pattern of regulation, increasing after 24 h and decreasing to basal levels after 48 h (Student’s *t*-test).

The previous experiments pointed to the importance of NMDA receptor activity in the spine formation process. In the early stages of postnatal cortex development, a large fraction of newly developed hippocampal synapses contain mainly NMDA receptors and are silent at the resting membrane potential (Kerchner and Nicoll, 2008). Thus, we next wondered whether the increase in synaptic contacts runs parallel to an upregulation of NMDA subunit expression. To this end, we used real-time PCR to quantify the expression of the NR1 subunit of the NMDA receptor in cultures stimulated with PTD4-PI3KAc or PTD4 as a control. The quantification was performed using mRNA from six different cultures from each of the two conditions after 24 and 48 h of treatment. The results showed a 322.8 ± 11.7% increase in the expression of the NR1 subunit after 24 h, only to decrease to 225.6 ± 11.6% 48 h later (**Figure 7**). Considering the relationship between glutamate receptor composition and spine size/maturation, we wanted to determine whether other members of the glutamate family of receptors were also upregulated. For this reason, we analyzed the expression of the GluR1 and GluR2 subunits. Interestingly, the expression of both subunits peaked at 160 ± 7.7% and 255.3 ± 24.7%, respectively, after 24 h, then descending to a basal level after 48 h (**Figure 7**). These results would indicate that PI3K activation produces a transcriptional upregulation of both NMDA and AMPA receptor subunits.

The activity-regulated cytoskeleton-associated protein (Arc) has been proposed as a regulator of spine morphology. Its expression is closely regulated by neuronal activity (Rao et al., 2006) and LTP consolidation (Messaoudi et al., 2007). Moreover, overexpression of Arc increases the density of thin spines in neuronal cultures (Peebles et al., 2010). Therefore, we next examined the expression profile of Arc in the same samples used for NMDA and AMPA receptor subunit quantification. Significantly higher levels of Arc expression (135.2 ± 10.8%) were detected 24 h after PI3K activation, which fell back to the basal levels after 48 h (**Figure 7**).

### PI3K OVEREXPRESSION INDUCES SMALL SPINE FORMATION IN CULTURED NEURONS

Our *in vivo* results suggest that PI3K favors the formation of new spines that are small in size. We next asked whether PI3K activation specifically promotes the formation of small-sized spines, or it just happens that new spines are always of small size. To answer this question, we used time lapse recordings of rat hippocampal neurons transfected with actin-GFP to determine the rate and type of spines induced by PI3K.

**FIGURE 8:**
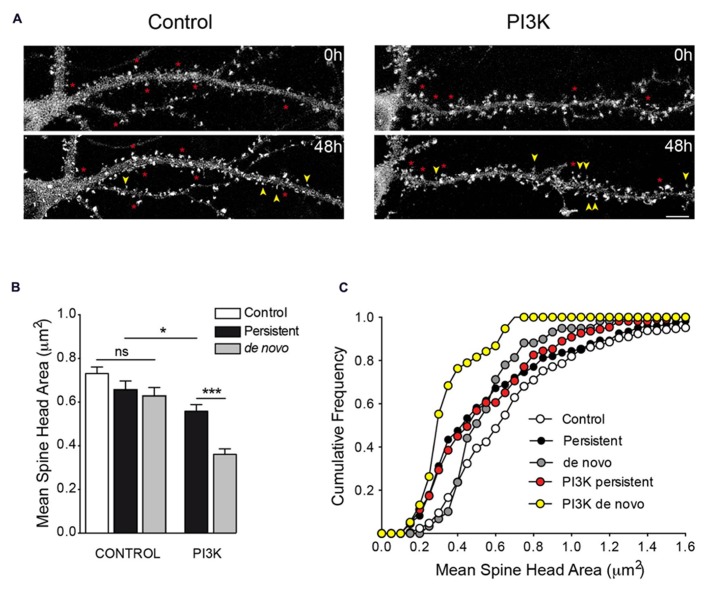
**Time lapse analysis of PI3K-induced spines. (A)** Representative frames from time lapse videos of hippocampal neurons transfected with actin-GFP in control and after 48 of PTD4-PI3KAc-treated (top and bottom pictures correspond to 19DIV and 21DIV, respectively). Yellow arrows indicate *de novo* spines, whereas asterisks indicate persistent spines. **(B)** Bar plot of the mean spine head areas. Under control conditions, there are no significant differences between persistent and *de novo* spines, although a tendency toward smaller values is evident. After PI3K activation, persistent spines have small size areas compared with control conditions, while the *de novo* spines were smaller compared to the persistent ones (Student’s *t*-test). **(C)** Cumulative frequency distributions indicate specific changes in spine head areas. In the absence of PI3K activation, persistent spines (closed circles) suffer a reduction of spine head when compared to control values (open circles), mostly affecting sizes below 1.0 μm (Kolmogorov–Smirnov, *p* < 0.001). The *de novo* spines (gray circles) exhibited smaller areas (statistically different from controls; Kolmogorov–Smirnov, *p* < 0.01), preferentially affecting spines larger than 0.4 μm. After PI3K treatment, the full range of spine size is affected and both distributions are characterized by the presence of small spines size population (*de novo*: yellow circles; persistent: red circles; Kolmogorov–Smirnov, *p* < 0.001).

First, the actual rate of spinogenesis in our own culture conditions was examined. Live images of dendrites were obtained at 19DIV. The same dendrites were imaged 48 h later (21DIV) and the number of spines were quantified, comparing these two stacks of pictures. In general, dendrites showed great variability in the percentage and proportion of changes. Nevertheless, of all the dendritic fragments analyzed, 45.5% exhibited a clear net reduction in spine density, while the remaining 54.5% showed an increase in spine density (**Table 4**). The percentage of change varied from dendrite to dendrite, but on average, the rate of reduction was 18 ± 4.5% and the percentage of increase was 28 ± 5.5%. Logically, when all dendrites where considered, an actual increase in spine number occurred as the cultures aged (**Figure 8A**).

**Table 4:** Summary of the analysis of spine turnover and the measurement of spine head areas in hippocampal neurons in culture.

Conditions	Number of dendrites	% of dendrites showing spines that disappear	% of dendrites showing *de novo* spines
Control	22	45.5	54.5
PI3K	23	17.4	82.6
	Mean head area (μm^2^) 19DIV*	Mean area (μm^2^) of persistent spines (48 h)	Mean area (μm^2^) of *de novo* spines (48 h)
Control	0.74 ± 0.04	0.66 ± 0.04	0.63 ± 0.04
PI3K	0.73 ± 0.04	0.56 ± 0.03	0.36 ± 0.02

Time lapse analysis of spine turnover in control conditions (11 cells in one culture) and after PI3K overactivation (10 cells in one culture). *19DIV correspond to control conditions. PTD4-PI3KAc was administered to the culture medium just after the start of time lapse recordings. Notice that in **Figure 8** both controls have been averaged together (white bar; 0.73 ± 0.03 μm^2^).

Next, we repeated the same experimental approach, but this time adding PTD4-PI3KAc to the culture media immediately after the first recordings at 19DIV. Spine turnover after PI3K activation evidenced values similar to those of the control conditions (21.9 ± 5.2% reduction and 22 ± 4.1% increment for dendrites with decreased and increased spine density, respectively); however, the proportion of dendritic fragments with a net increase in spine density augmented by 82.6% (**Table 4**). Although activation of PI3K does not raise the actual rate of spine formation, it actually increased the number of dendrites, which began an active process of spinogenesis.

We next performed a morphological study measuring spine head areas. Using time lapse video recordings, the population of spines was divided between persistent (permanent spines during the 48 h interval) and *de novo* spines (**Figure 8A**). The results indicated that under control conditions, the spine head areas were similar during the 48-h experimental period; the *de novo* spines exhibited a slight tendency toward a reduced head area, although this was not statistically significant when compared to the control or persistent spine areas (**Figure 8B**). The picture was quite different after PI3K activation; in this case, 48 h later, the averaged head areas were significantly smaller than in the controls, moreover, the *de novo* spines exhibited the smallest areas of all (**Figure 8B**). In addition, we used cumulative frequency distributions to analyze the trends in these areas (**Figure 8C**). The analysis demonstrated a general tendency toward smaller head areas in all conditions, with the clearest shift in size affecting primarily the *de novo* PI3K-induced spines. From these distributions, we can conclude that PI3K activation favors the formation of new spines with a small head area and reduces the area of preformed spines.

### PI3K OVEREXPRESSION INCREASES SPINE DENSITY IN THE Tg2576 MICE MODEL OF ALZHEIMER’S DISEASE

Up to this point, our data indicated that PI3K is able to modify spine number *in vivo* in the hippocampus and in cultured neurons. We then wondered whether PI3K activation would also have a spinogenic action in a context of neurodegeneration, where cells are known to be vulnerable to losing spines. With this aim, we finally studied spine density in 6-month-old Tg2576 transgenic mice, an age when contextual memory deficits and spine loss on hippocampal pyramidal neurons is already evident (Hsiao et al., 1996; Lanz et al., 2003).

**FIGURE 9:**
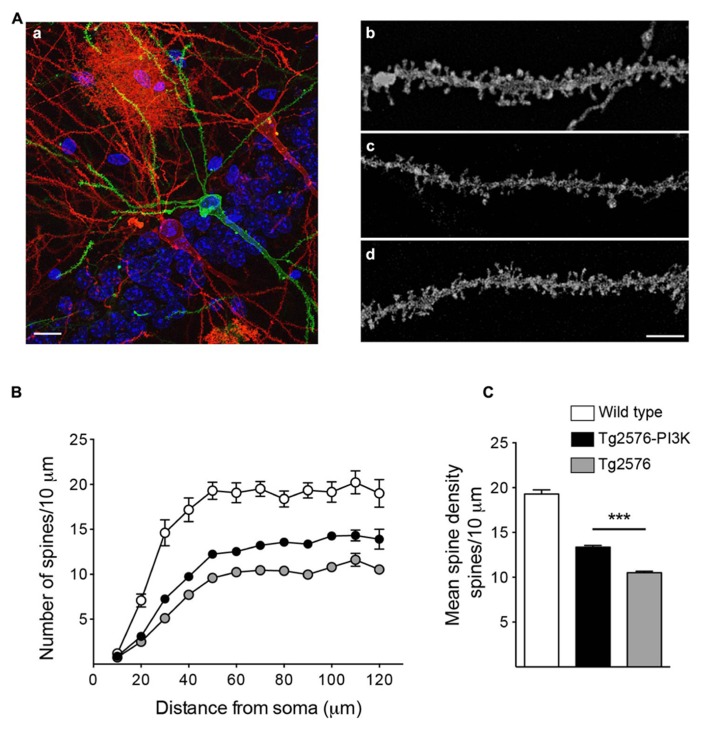
**PI3K increases spine density in a Tg2576 mice model. (A)** (a) Example of biolistic stain, the image shows an area from the basal CA1 region of a 6-month-old Tg2576 mouse.Three hippocampal neurons appear stained with DiI (red) or DiO (green). The stratum pyramidale nuclei appear in blue (DAPI). An astrocyte, red area in the top middle side of the picture was also labeled. Scale bar: 10 μm (a). Representative dendritic segments for Wild-type (b), Tg2576 (c), and Tg2576 injected with PTD4-PI3KAc (d). Scale bar = 3 μm. **(B)** Number of spines along basal dendrites in neurons from wild-type (open circles), Tg2576 (gray circles), and PTD4-PI3KAc-treated Tg2576 mice (closed circles). The values were statistically different between 30 and 120 μm from the soma (two-way ANOVA, *p* < 0.001; significant levels are not included for clarity). **(C)** Mean spine densities considering all values 60 μm or more away from the soma (a distance at which the number of spines have reached a plateau). The number of spines in Tg2576 mice (10.5 ± 0.16, 50 dendrites from four animals) is significantly reduced compared to the wild-type (19.3 ± 0.47, 30 dendrites from three mice). Tg2576 injected with PTD4-PI3KAc exhibited an intermediate value (13.8 ± 0.18, 50 dendrites from six animals), suggesting that overactivation of PI3K partially counteracts the loss of spines in Tg2576 mice.

To evaluate the distribution of spines along primary basal dendrites, we used a stereotaxic procedure similar to that used in rats. In this case, 20 μg of PTD4 or PTD4-PI3KAc (Tg2576 and Tg2576 PI3K, respectively) were injected into the lateral ventricle of the transgenic mice. At 72 h post-injection, the animals were sacrificed and their brains removed for vibratome sectioning and diolistic labeling (**Figure 9A**). In parallel, a cohort of 6-month-old wild-type mice, not subjected to stereotaxis, was used as a wild-type control group. As shown in **Figures 9A–C**, Tg2576 mice exhibited a clear loss of spines, as compared to wild-types, while Tg2576 mice treated with PTD4-PI3KAc evidenced a significant increase in the number of spines, as measured between 30 and 120 μm from the soma (**Figure 9C**). On average, WT, Tg2576 and PI3K-injected animals had, respectively, 19.3 ± 0.47, 10.5 ± 0.16, and 13.8 ± 0.18 spines every 10 μm. Thus, our results confirmed that the spinogenic activity of PI3K is also conserved in this neurodegenerative background.

## DISCUSSION

Three are the main findings of our studies: (i) PI3K activation modulates spine formation, (ii) PI3K activation favors the formation of small spines, and (iii) the structural feature that correlates with better learning abilities is the average head area of the spines.

### STRUCTURAL REMODELING AFTER PI3K OVERACTIVATION

There is plenty of evidence that associates learning with modifications in neuronal structural plasticity (Holtmaat and Svoboda, 2009; Caroni et al., 2012). For instance, exposure to a rich environment promotes spine formation in the hippocampus (Gogolla et al., 2009; Bednarek and Caroni, 2011). By using *in vivo *microscopy in mice, it has been found that fear conditioning and extinction produce opposite effects on spine formation on pyramidal neurons in the frontal association cortex. Thus, fear conditioning by pairing an auditory cue with a foot-shock led to spine elimination, while the fear extinction caused new spines to grow (Lai et al., 2012). In this sense, it is known that mice exposed to the fear contextual conditioning increased spine density on apical and basal dendrites of pyramidal neurons in the CA1 hippocampus region, while pyramidal neurons from the anterior cingulate cortex exhibit a decrease in spine number (Restivo et al., 2009). In agreement with Restivo and colleagues, we have observed an increase of spine density**in CA1 after CFC. In our study, PI3K activation does not upregulate spine density over the CFC test, suggesting that CFC induces a ceiling effect on this parameter. From this observation, it was evident that a mere change in spine density would not account for the contextual memory differences observed between both groups; the analysis of spine head area indicates that CFC favors a shift toward larger areas, whereas PTD4-PI3KAc injection favored small-sized spines, which are known to be particularly plastic and related to learning (Bourne and Harris, 2007; Kasai et al., 2010). From this set of data, we conclude that it is the change in size distribution which correlates with the improved learning abilities after PTD4-PI3KAc injection. Furthermore, the *in vitro* experiments on actin-GFP transfected neurons, suggests that overactivation of PI3K regulates both the formation of new small spines and the remodeling of persistent ones. We hypothesized, that in the hippocampus of an animal subjected to a CFC protocol, spine density might already be saturated, and PI3K activation would preferentially counteract the effects of CFC over spine size.

### SPINE SIZE AS A KEY ELEMENT LINKED TO FUNCTIONAL ATTRIBUTES

Classically, spines have been grouped into three categories: stubby, thin, and mushroom-shaped (Peters and Kaiserman-Abramof, 1970; Harris et al., 1992). This distribution depicts a static picture of spines. To avoid this bias, we chose to consider spine head areas as a part of a continuous distribution, arbitrarily dividing spines between small and large with respect to the mean area value, following a classification method similar to that described by Peebles et al. (2010) and Dumitriu et al. (2010). Disregarding the underestimation of spine density resulting from the use of Golgi staining and our technical approximation to measure spine size, controls, and experimental conditions were analyzed according to the same criteria, therefore constituting a homogeneous population.

PTD4-PI3KAc injection induces a transient and reversible increase of spine density, a degradation of the peptide can easily account for this result. In naive animals after PI3K injection, spine head areas did not change during the first 72 h, and only a slight enlargement after 96 h occurred; although the overall spine head areas remains small when compared with the changes induced by CFC. It is possible that after 96 h of PI3K activation *in vivo*, a process of stabilization of the new spines would take place that can account for the shift in spine size distribution.

Similarly, in hippocampal cultures, spine density was upregulated after PI3K activation following a comparable time window; nevertheless, inhibition of PI3K activity induces a partial reduction of spine density. Phosphoinositides, as second messengers, have a pivotal role regulating the activity of several actin binding proteins and have been implicated in spine formation (Ueda and Hayashi, 2013) and in the regulation of AMPA receptor presence at the postsynaptic density (Arendt et al., 2010). Altogether, the evidences suggest that a basal level of PI3K activity must be required to maintain spine structures.

The morphometric analysis by spine types also reveal that small spines are always the more prone to change, while large spines tend to be stable. In naive animals large spines did not change in size and although the CFC test induces an overall enlargement of all spine types, the most dramatic changes occur in the small size group. Furthermore, our results indicate that PI3K activation can induce structural remodeling by favoring small head spine morphology. These changes observed are compatible with the high turnover and plasticity that characterize the small spines (Yasumatsu et al., 2008; Kasai et al., 2010).

After PI3K activation, what signaling pathway could explain the changes in spine density and area? We and others have demonstrated that PI3K activation promotes changes in dendritic morphology and formation of synaptic contacts in insects and mammals through an Akt-mTOR-dependent mechanism (Mart’i, 2006; Cuesto et al., 2011; Acebes et al., 2011; Urbanska et al., 2012). Furthermore, neurons lacking Pten exhibit neuronal hypertrophy and high spine density (Kwon et al., 2006; Luikart et al., 2011), and inhibition of GSK3, a downstream element of the PI3K-Akt pathway, increases spine density in cultured neurons (DaRocha-Souto et al., 2012). As further support for the involvement of this pathway, insulin promotes spine formation through a PI3K-Akt-mTOR-dependent mechanism, whereas Rac1 inhibition by siRNAs counteracts these effects, evidencing a connection between PI3K, spine formation and actin cytoskeleton remodeling (Lee et al., 2011; Rácz and Weinberg, 2013).

### NMDA ACTIVITY CORRELATES WITH THE FORMATION OF NEW SPINES

NMDA receptors predominate in small spines, while enlargement and stabilization correlates with AMPA receptor insertion (Wang and Zhou, 2010). The gene expression profile indicates that after PI3K activation, the NR1 subunit was upregulated for 48 h, while GluR1 and GluR2 subunits peaked at 24 h and reached basal levels after 48 h, suggesting that the new born spines would be enriched in NMDA receptors.

NMDA activation is a prerequisite for a synaptic/spine contact formation (Rao and Craig, 1997; Shi et al., 1999). NMDA receptors are blocked by extracellular magnesium at resting potential (Johnson and Ascher, 1999). Despite presence of magnesium on culture media, spontaneous depolarizations of the postsynaptic membrane would allow NMDA opening. To ensure that NMDA activation was totally inhibited, APV, a competitive blocker of NMDA receptor was employed. In the presence of the blocker, the PI3K-spinogenic effect was inhibited, therefore demonstrating the need of NMDA activation for the formation of new spines.

The immediate-early gene Arc, favors the formation of new small-sized spines in hippocampal cultures (Peebles et al., 2010). PI3K activity upregulated Arc transcription levels, even after 24 h of peptide addition, favoring the formation of small spines. In summary, the genes expression profile also supports the appearance of small spines after PI3K activator peptide treatment.

### ELECTROPHYSIOLOGY RECORDINGS SUGGEST THAT NEW SPINES ARE FUNCTIONAL

Due to the experimental design, higher spine density was only patent *in vivo* after 72 to 96 h, nevertheless as early as 24 h after PTD4-PI3K injection, chronic recordings at the dorsal hippocampus evidenced a clear potentiation of spontaneous LFPs that reached maximum levels 48 h later and remain potentiated during 144 h. Although we cannot underestimate a functional potentiation of synaptic transmission, the increase in field potential size during the 72–96 h interval suggests that new spines are functional.

The electrophysiological study was limited to previously characterized LFP generators at the CA1 level (Benito et al., 2013). The data presented from st. rad, and lac/mol correspond to virtual LFPs, i.e., generator-specific LFPs reconstructed for each of the afferent pathways making synaptic contact in these dendritic portions. Although the recording array covers all hippocampal layers from the alveus down to the dentate gyrus, only the ipsilateral CA3 Schaffer input is regular enough to allow the individual waves being quantified. Except for the mixture of pathways contained in the lac/mol LFP generator, other local or extrinsic pathways ending in the CA1 (e.g., septal and contralateral CA3 inputs, and inputs from several interneuron subnetworks) do not fulfill the spatial and temporal criteria required to generate sizable spontaneous population activity (Benito et al., 2013). Therefore, their possible changes, if any, cannot be examined by LFPs. 

Since LFPs reflect the temporal structure of the output of afferent populations, the stability of the frequency and duration of the spontaneous microfield potentials indicates that the changes in amplitude are due to a modification of postsynaptic currents. It should be noted that microfield potentials are generated by the synchronous activation of multiple CA3 neurons (Fernández-Ruiz et al., 2012b), representing the summation of thousands of synapses. Thus, a change in the density of postsynaptic receptors or an increase in the number of spines would best explain these observations. Even though, a functional potentiation during the first hours is an interesting possibility, since PI3K has been implicated in LTP late phase stabilization (Sanna et al., 2002) and AMPA receptor traffic (Arendt et al., 2010; Jurado et al., 2010; Acebes and Morales, 2012). Future experiments studying synaptic plasticity would clarify this point.

### PI3K SPINOGENIC EFFECT IN Tg2576 MICE

Memory impairments correlating with the loss of synapses and spines occur during neurodegenerative diseases and aging (Hao et al., 2006; Dumitriu et al., 2010; Penzes et al., 2011). In Tg2576 mice, a decrease in spine density in basal dendrites of CA1 pyramidal neurons takes place as early as 4.5 months of age (Lanz et al., 2003). Around the same age, granule cells from the dentate gyrus display a characteristic loss of spines that correlates with a significant impairment of LTP on slices and with the contextual memory deficits observed in transgenic animals (Jacobsen et al., 2006). The fact that PI3K overactivation by the peptide is able to produce a meaningful increase in spine density indicates that regulation of PI3K activity also counteracts the loss of dendritic spines caused by neurodegeneration, suggesting that modulation of the PI3K pathway might be a therapeutic way to treat neurodegenerative diseases.

A body of evidences suggests that structural plasticity is an integrated aspect of learning and memory (Caroni et al., 2012). Hormone level, age, stress, an enriched environment or a CFC protocol, produce global alterations in spine turnover. This broad alteration would probably facilitate the dynamics of a specific fraction of spines that would eventually retain the structural memory trace (Caroni et al., 2012). In a similar way, we hypothesize that a global PI3K activation favors a destabilization of the spine structure, causing the spine to enter into a plastic state, providing the potential molecular substrate for a specific learning.

## AUTHOR CONTRIBUTIONS

Lilian Enriquez-Barreto conducted the research. Germán Cuesto and Carmen Sandi, the CFC test experiments. Oscar Herreras, Antonio Fernández-Ruiz and Gonzalo Martín-Vázquez, the electrophysiology recordings. Diego Ruano and Elena Gavilán, the mRNA expression analysis and Nuria Dominguez-Iturza the Tg2576 spine density counting. Lilian Enriquez-Barreto and Miguel Morales wrote the manuscript. Miguel Morales supervised the project and edited the manuscript.

## Conflict of Interest Statement

The authors declare that the research was conducted in the absence of any commercial or financial relationships that could be construed as a potential conflict of interest.
